# Coccidioidomycosis Masquerading as Malignancy: A Case of Mediastinal Mass and Pericardial Effusion in an Immunocompetent Jamaican Male

**DOI:** 10.1155/crdi/5512772

**Published:** 2026-02-17

**Authors:** Samuel B. Governor, Maira Zaidi, Prakash Nepali, Yulia Grigorova, Amali Baissari, Richard Toussaint, Mariam Dabaghyan, Alberto Melendez-Garcia, Rita Jammal

**Affiliations:** ^1^ Internal Medicine Department, St. John’s Episcopal Hospital, Far Rockaway, New York City, New York, USA, ehs.org; ^2^ Internal Medicine Department, Ross University School of Medicine, Bridgetown, Barbados, rossu.edu

## Abstract

Coccidioidomycosis rarely presents as a lung mass, hypereosinophilia, and disseminated pericarditis with pericardial effusion. Even rarer is a case that presents with the constellation of all these findings. We present a case of coccidioidomycosis in an immunocompetent young Jamaican male with all these features, set against an interesting epidemiological context of a likely exposure to coccidioidomycosis spores during an on‐foot crossing of the Mexican border into Texas. His treatment course was complicated by declining liver and kidney function due to triazole antifungal therapy, necessitating titration of these doses to tolerable levels. This case highlights the importance of a detailed travel history in unraveling infectious disease diagnoses and underscores the need for high clinical suspicion for coccidioidomycosis, even in patients outside of known endemic regions.

## 1. Introduction

Coccidioidomycosis, commonly known as Valley fever, is a fungal infection caused by inhaling soil‐dwelling spores [1]. It is endemic to regions of California, New Mexico, Arizona, and Texas in the United States [2]. The disease is caused by two genetically distinct species: Coccidioides immitis (primarily prevalent in California) and Coccidioides posadasii (found mostly outside of California) [3]. The incubation period typically ranges from one to 3 weeks [4], and the clinical presentation varies from asymptomatic infection to severe manifestations, including acute pneumonia and disseminated disease.

The initial presentation often mimics community‐acquired bacterial pneumonia [3], leading to missed diagnoses, particularly in nonendemic areas where clinical suspicion is lower. Diagnosis is primarily achieved through serologic testing, which often takes several days to yield results [3]. Coccidioidomycosis is more likely to disseminate in immunocompromised individuals, such as those with human immunodeficiency virus/acquired immunodeficiency syndrome (HIV/AIDS), organ transplants, the elderly, and those traveling to or residing in endemic areas [5]. However, disseminated disease can also occur in individuals without obvious immunocompromising conditions, particularly in certain ethnic groups, including Hispanics, African Americans, and Filipinos [5].

While dissemination commonly involves the skin, bones, and central nervous system, in rare cases, the disease may present with mass‐like lesions mimicking malignancy, further complicating diagnosis [5–8]. In an even rarer subset of cases, pericardial involvement can occur, sometimes leading to pericardial effusion. Chan et al. identified 23 cases of coccidioidomycosis with pericardial involvement in the literature [9], while a study conducted by Heidari et al. identified only 5 cases of pericardial involvement out of 1771 coccidioidomycosis cases [10].

We report a case of an immunocompetent young Jamaican male and chronic smoker who arrived in the United States by crossing the Mexican border into Texas on foot and later developed pneumonia‐like symptoms. His delayed presentation to the emergency department likely contributed to the development of disseminated coccidioidomycosis, presenting as pericarditis with pericardial effusion.

The unique constellation of symptoms in this case—including pericarditis with pericardial effusion, incidental discovery of the effusion during a liver ultrasound, hypereosinophilia, mediastinal lung mass causing dysphagia, and tracheal obstruction—and the patient’s epidemiological context of exposure make this case exceptionally notable and distinct from previously reported cases in the literature.

## 2. Case Presentation

The patient was a 44‐year‐old male with no significant past medical history. He was a marijuana and cigarette smoker who presented to the emergency department with midsternal chest pain, fever, productive cough, and shortness of breath that began 2 weeks prior. These symptoms were associated with difficulty swallowing solid food, nausea, and anorexia. The patient reported an unintentional weight loss of approximately 6‐7 pounds over the past 2 weeks due to reduced oral intake.

He had recently emigrated from Jamaica and spent approximately 1 month in Mexico before crossing the Mexican border into Texas on foot. He denied vomiting, diarrhea, allergies, or a history of treatment for helminthic infections. The patient had been self‐treating with ampicillin at home for 2 weeks without improvement and presented to the emergency department due to persistent fever and a predominantly dry cough, occasionally producing clear sputum.

**FIGURE 1:**
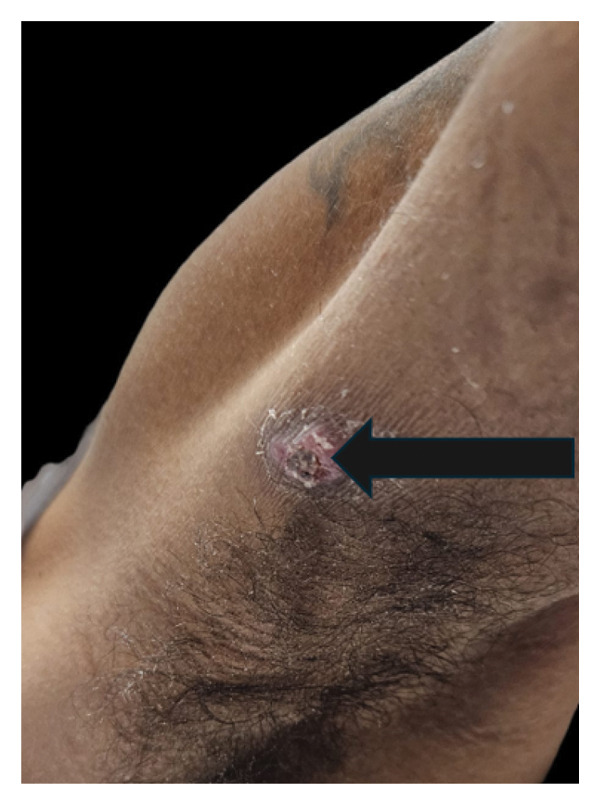
Ulcerated nodule in the right axilla, indurated and chronic‐appearing, circular with irregular borders, central ulceration, and surrounding induration.

On physical examination, there was an ulcerated skin nodule in the right axilla (see Figure 1) and decreased breath sounds on the right side, without crackles or wheezing. He was febrile, tachycardic, and hypotensive, with an oxygen saturation of 97% on room air.

**FIGURE 2:**
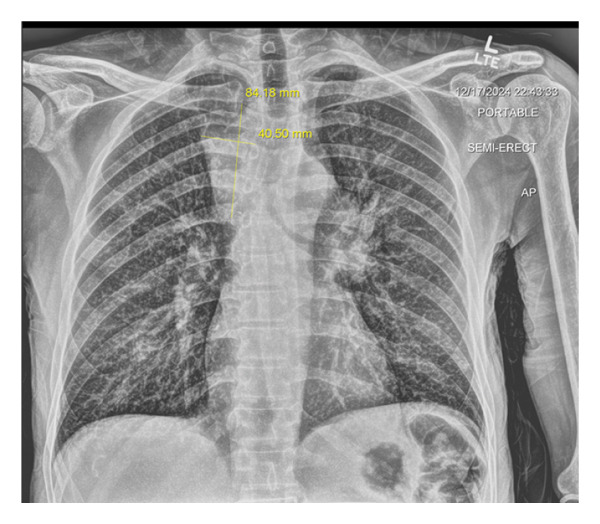
Chest x‐ray showing the mediastinal mass at the right paratracheal region. An AP view of the chest x‐ray in Figure 2 shows no pleural effusion, mild interstitial prominence versus crowding of the vascular markings, and increased opacification along the right paratracheal region. Differential diagnosis included atelectasis versus infiltration versus mass.

Laboratory studies were notable for moderate normocytic anemia with normal red cell distribution width (RDW), leukocytosis with marked eosinophilia and thrombocytosis, and hyponatremia, see Table 1. A chest radiograph (x‐ray) revealed a suspected mediastinal mass and bilateral lymphadenopathy (see Figure 2). A contrast‐enhanced computed tomography (CT) scan of the chest was recommended for further evaluation, revealing a mediastinal superior mass most consistent with malignancy, possibly representing primary and/or metastatic disease. The scan showed a large heterogeneous mass in the superior mediastinum above the carina causing compression of the trachea, esophagus, and the left common carotid artery slightly narrowed due to mass effect. The mass measures 48.98 × 47.28 × 90.53 mm^3^, see Figures 3(a), 3(b), and 3(c).

**TABLE 1:** Laboratory findings at presentation with corresponding reference ranges.

Lab names	Labs	Units	Normal interval
White blood cells (WBCs)	13.4	10^3^/UL	5.2–12.4
Hemoglobin (Hgb)	11.5	G/DL	14–18
Red cell distribution width (RDW)	13.8	%	11.5–14.5
Eosinophils (Eos %)	15.7	%	0.0–7.0
Platelet counts (Plt)	488	10^3^/UL	130–400
Sodium (NA)	130	Mmol/L	137–145

FIGURE 3(a) CT scan with contrast showing the width and anterior–posterior measurement of the mediastinal mass (48.98 × 47.28 mm) and length of 90.53 mm (b). The mass, therefore, measures 48.98 × 47.28 × 90.53 mm^3^. (b) CT scan with contrast showing the cranial‐caudal length of the mediastinal mass measuring 90.53 mm. (c) CT scan with contrast of the mediastinal mass compressing upon the trachea and the esophagus.

**Figure:**
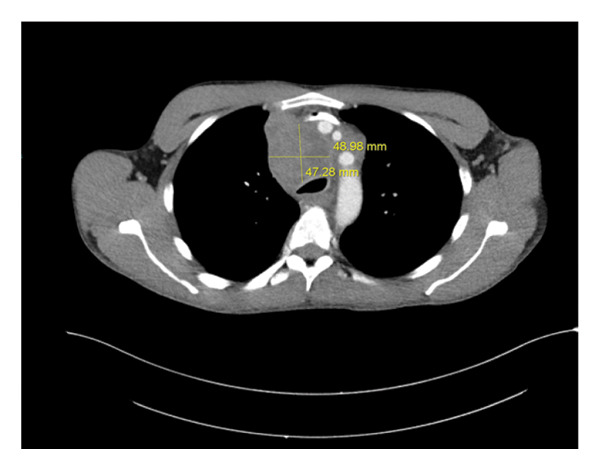
(a)

**Figure:**
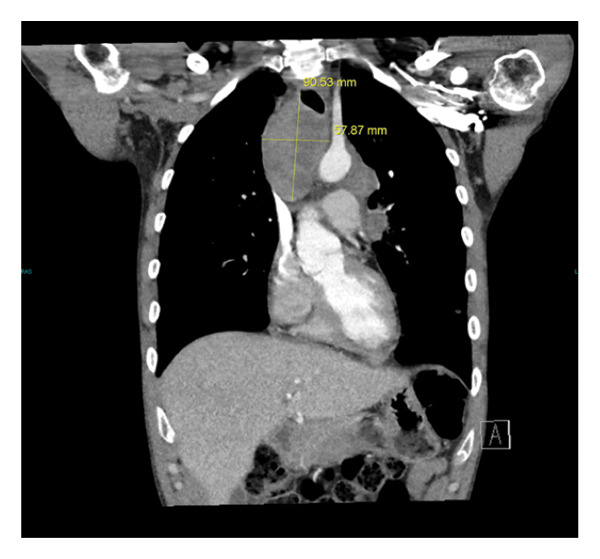
(b)

**Figure:**
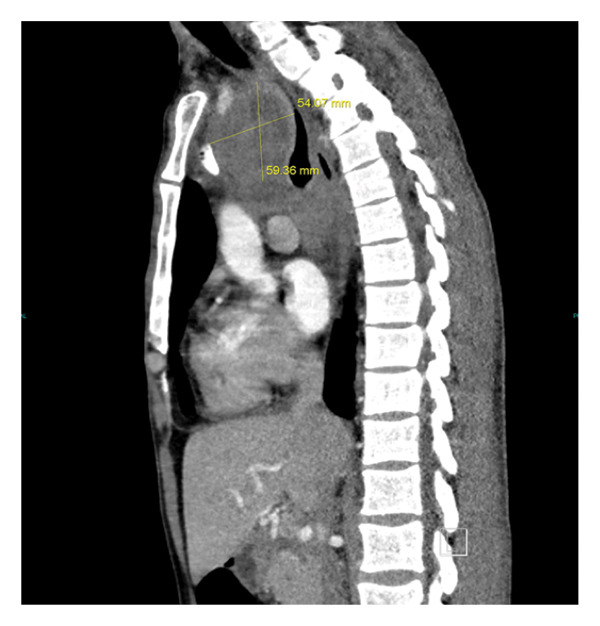
(c)

Differential diagnoses included lung malignancy, metastatic disease, tuberculosis, and infectious causes such as fungal, protozoan, or bacterial pneumonia.

Diagnostic workup was performed accordingly. Blood and urine cultures showed no bacterial growth. Stool studies for ova and parasites were negative for helminths and protozoa. Venereal disease research laboratory (VDRL) testing for syphilis and HIV testing were negative. Quantiferon testing for tuberculosis was negative. Serologic studies for coccidioidomycosis—though significantly delayed—returned a positive result. Tumor markers, including carcinoembryonic antigen (CEA) and prostate‐specific antigen (PSA), were within normal limits. A brain CT scan showed no acute intracranial findings. A whole‐body bone scan revealed no evidence of metastatic disease.

**FIGURE 4:**
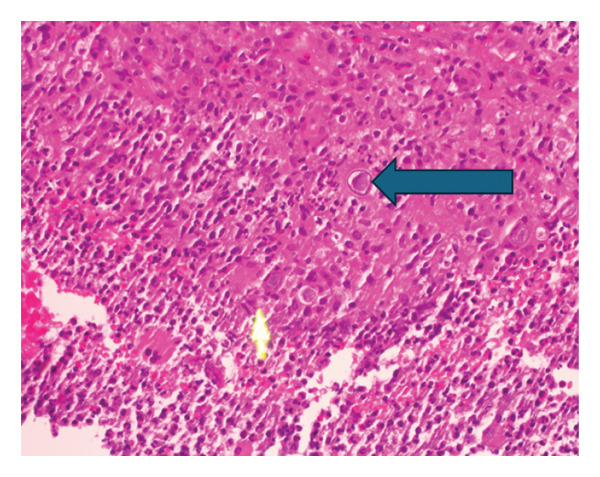
Granulomatous inflammation and fungal elements consistent with Coccidioides species with PAS stain strongly positive.

A CT‐guided biopsy of the mediastinal mass showed granulomatous inflammation and fungal elements consistent with Coccidioides species, with a strongly positive Periodic Acid‐Schiff (PAS) stain, see Figure 4. However, fungal culture was canceled due to an insufficient number of cells.

The patient was initially treated with liposomal amphotericin B (315 mg IV daily), which was later switched to oral fluconazole (800 mg daily) due to worsening kidney function. He responded clinically, reporting symptomatic improvement within approximately 1 week. However, he subsequently developed adverse effects—elevated liver enzymes, nausea, and anorexia—likely attributable to fluconazole. The dose was reduced to 400 mg oral daily, but liver enzyme levels continued to rise.

Fluconazole was held due to persistent transaminitis and the patient’s complaints of chest pain and shortness of breath. A liver ultrasound was performed, during which an incidental finding of pericardial effusion suggestive of pericarditis was noted. The patient was transferred to another hospital for a higher level of care and underwent a pericardial window procedure. Following the procedure, he was restarted on fluconazole 200 mg oral daily, which he was tolerating at the time of this report.

## 3. Discussion

The rarity of lung mass presentations in coccidioidomycosis can lead to missed diagnoses and delays in treatment. Case studies like ours are essential for raising clinical suspicion of coccidioidomycosis, particularly among immigrants or travelers from endemic regions. A detailed travel history is the key to making this diagnosis. However, it is equally important to inquire about the mode of travel. In our case, the patient crossed the Mexican border into Texas on foot—likely increasing his risk of exposure to Coccidioides arthroconidia spores, which reside in the soil. Thus, while obtaining a thorough travel history is crucial, physicians should also consider asking unconventional questions, such as how the patient traveled through endemic areas, as certain methods may increase the risk of inhalation exposure.

The patient reported leaving Mexico in October and developing symptoms in November, corresponding to an incubation period of approximately 3 weeks [4]. This timeline aligns with the known incubation period for coccidioidomycosis and supports the likelihood that exposure occurred during his journey across the border on foot.

Given the diagnostic challenges and delays often associated with coccidioidomycosis, patients presenting with a constellation of symptoms—including constitutional signs, pulmonary findings, eosinophilia, and skin lesions—should prompt a high index of suspicion. In our case, the patient also developed a pericardial effusion, which was not evident on initial imaging and was discovered incidentally over a week later. This highlights the limitations of standard imaging and reinforces the importance of early cardiac evaluation when dissemination is suspected.

In endemic areas—or in patients with relevant travel history—empirical antifungal therapy may be justified while awaiting definitive diagnostic confirmation. Early initiation of treatment may reduce the risk of extrapulmonary dissemination. Our patient was treated with liposomal amphotericin B, followed by oral fluconazole. This treatment approach is consistent with guidelines for severe or disseminated coccidioidomycosis, which recommend initial therapy with amphotericin B followed by extended triazole therapy for at least 6–12 months.

However, the potential for adverse effects—including liver and kidney toxicity—must be recognized. In our case, the patient poorly tolerated fluconazole at doses of 800 mg and 400 mg daily, developing hepatotoxicity. Clinicians should be open to dose adjustments based on tolerance. Following the patient’s surgical pericardial intervention, fluconazole was restarted at 200 mg PO daily, which he was able to tolerate at the time of this report.

### 3.1. Limitations

Although the diagnosis in our case was confirmed histopathologically using a biopsied sample, we were unable to perform a culture to isolate the Coccidioides species responsible for this presentation. Future cases should ensure that biopsied specimens are adequate for both histopathological and microbiological analysis—including fungal culture—to enhance diagnostic accuracy and species identification.

## 4. Conclusion

Coccidioidomycosis can present as a lung mass with symptoms such as nausea, vomiting, unintentional weight loss, and dysphagia—often mimicking lung cancer. Therefore, the differential diagnosis of lung masses should include coccidioidomycosis, particularly in immigrants, travelers from endemic regions, or residents of those areas. This infection can disseminate to the pericardium and cause pericardial effusion, even in immunocompetent individuals.

Early recognition is essential to prevent diagnostic delays and improve clinical outcomes. Clinicians must maintain a high index of suspicion for this infection in endemic settings and among travelers, incorporating thorough travel histories—including modes of travel—and appropriate diagnostic tools in their evaluation.

## Funding

No funding was received for this manuscript.

## Conflicts of Interest

The authors declare no conflicts of interest.

## Data Availability

Data sharing is not applicable to this article as no datasets were generated or analyzed during the current study.
